# Novel biomarkers for subtle myocardial involvement in type I diabetes mellitus

**DOI:** 10.1097/XCE.0000000000000240

**Published:** 2020-11-19

**Authors:** Sonia A. El-Saiedi, Mona H. Hafez, Yasser M. Sedky, Sahar A. Sharaf, Mona S. Kamel, Antoine F. AbdelMassih

**Affiliations:** a Division of Pediatric Cardiology, Pediatrics Unit; b Department of Clinical Pathology; c Fellow of Pediatric Cardiology, Pediatrics Unit, Cairo University, Cairo, Egypt

**Keywords:** biomarkers, brain natriuretic peptide, plasma cardiotrophin left ventricular dysfunction, type 1 diabetes mellitus

## Abstract

**Background:**

Evaluation of certain biomarkers could be used to predict left ventricular (LV) and right ventricular (RV) function impairment in children with type 1 diabetes mellitus. The aim of this study was to determine the best cardiac biomarker for prediction of diabetic cardiomyopathy.

**Methodology:**

This study was designed as case-control study. A total of 55 children with type 1 diabetes mellitus (group/G1) and 55 healthy controls (G2) were subjected to echocardiography including 3D-Speckle Tracking Echocardiography and tissue Doppler imaging for assessment of RV and LV systolic and diastolic functions. As well as HbA1c, troponin I, brain natriuretic peptide (BNP), plasma cardiotrophin (CT-1), activin-A, transforming growth factor-β, and human insulin-like growth factor binding protein-7 (IGFBP-7) measurements.

**Results:**

Diabetic patients showed RV and LV systo-diastolic dysfunction compared to controls, the best predictor of LV systolic dysfunction was CT-1 (sensitivity: 69%, while IGFBP-7 was found to be the best predictor of RV systolic dysfunction (sensitivity: 63%). BNP was found to the best predictor of diastolic RV and LV dysfunction (sensitivity: 82% for both).

**Conclusion:**

CT-1 has proven to be a diagnostic superiority in LV systolic dysfunction whilst BNP continues to prove every day through our study and through many others that it is the chief marker of diastolic dysfunction and HFpEF. This potential accuracy and the increasing availability of BNP in the outpatient setting make it clear that it should be used as a screening test for diabetic patients.

## Introduction

Diabetes mellitus is an established risk factor for cardiovascular events and the development of heart failure. Various independent investigators have shown that in diabetic patients there is extensive impairment in left ventricular (LV) functions before the clinical signs of congestive heart failure become manifest. Diastolic dysfunction has been defined as the earliest sign of diabetic myocardial disease to occur before systolic impairment. This isolated diastolic impairment has been alternatively termed heart failure with preserved ejection fraction (HFpEF) [1,2].

Although echocardiography is the most useful non-invasive diagnostic method for evaluating systolic and diastolic dysfunction, the prognostic value of echocardiography is much less superior than its diagnostic potency. Heart failure results from a complex interplay between genetic, neurohormonal, inflammatory, and biochemical changes acting on cardiac myocytes. The aforementioned facts point at the possible role of biomarkers in the earliest detection and follow-up of changes occurring at the cellular level even in the absence of any alteration in echocardiographic indices [3].

At the practice level, some biomarkers have established role in daily life; notably, cardiac troponins are the chief markers of myocardial infarction (MI). Brain natriuretic peptide (BNP) has proven a diagnostic accuracy for chronic left ventricular (LV) dysfunction rather than acute. It takes longer time to rise in the context of acute MI and lasts longer than cardiac troponins. Therefore, its use as marker of acute myocardial ischemia would be a part of multimarker strategy incorporating its role together with cardiac troponins [4].

Also, BNP has proven a greater superiority in detection of diastolic dysfunction earlier than systolic dysfunction, which raises the need for other markers of systolic function that can add to the diagnostic accuracy of cardiac biomarkers in the context of prediction of heart failure [5].

The aim of this work is to test several new biomarkers such as BNP, plasma cardiotrophin (CT-1), human activin A (ACV-A), human transforming growth factors β1 (TGF-β1), human insulin-like growth factor binding protein-7 (IGFBP-7) in the early detection of systolic and diastolic dysfunction in the context of diabetes mellitus.

## Materials and methods

### Study subjects

This case-control cross-sectional observational study was conducted from September 2018 to September 2019. A total of 60 patients with chronic kidney disease were enrolled from Nephrology unit at Cairo University Children Hospital. Exclusion criteria included arrhythmia, congenital or acquired heart disease as well as obesity that may impair image acquisition, or any known comorbidities from diabetes.

We also enrolled 60 age- and sex-matched healthy controls (group 2).

Written and informed consent was obtained from all the study participants. The study was approved by the local ethics committee of Cairo University.

### Study methods

#### History taking and physical examination

Patients were subjected to a full clinical history including data from age, sex, onset of diabetes, diabetes duration, daily insulin requirements, and blood and urine readings. Physical examination by endocrinologist included a blood pressure (BP) assessment Anthropometric data were collected including weight, height, and body surface area. Glycated hemoglobin (HbA1C), high-density lipoprotein (HDL), low-density lipoprotein (LDL), triglycerides were retrieved from the patients’ files over the last year (done every 3-month interval) and were averaged to ensure reflecting the continuous monitoring of glycemic and lipidemic control of the patients.

*Echocardiography:* According to the guidelines of the American Society of echocardiography [6,7].

*For diastolic function:* Conventional Doppler and tissue Doppler (TDI) have been used to determine the LV E/E′ ratio: the ratio of early mitral inflow velocity to average early diastolic velocities of the basal septum and mitral annulus.

#### Real-time 3D echocardiography

For the LV: Full-volume acquisition of the LV will be obtained by harmonic imaging from the apical approach. All datasets were analyzed offline using commercially available software (4D Auto LVQ; GE-Vingmed, Horten, Norway). The software automatically will identify the LV cavity endocardial border in 3D. The operator will perform all the necessary adjustments manually in order to correctly place the endocardial border. After the adjustments, software will provide the LV global longitudinal strain (GLS).

For the right ventricle (RV): 3DE RV datasets were digitally analyzed offline using the commercial software TomTec RV Function 2.0 (Imaging Systems GmbH, Unterschleissheim, Germany) to generate RV GLS.

#### Cardiac biomarkers

Blood was collected into EDTA tubes and processed immediately and the following biomarkers were tested by ELISA as follows:

(1) BNP concentrations were determined by the ELISA Kit (Elecsys; Roche Diagnostics, Basel, Switzerland) with an assay range of 6–400 pg/mL [8].

(2) CT-1 by the ELISA Kit (NUNC Maxisorp, Nunc, Denmark) with an assay range of 0.5–200 ng/L [9].

(3) Human activin A (ACV-A) by the ELISA Kit (Ansh Laboratories, Webster, Texas, USA) with an assay range of 8–350 pg/mL [10].

(4) TGF-β1 by the ELISA Kit (Quantikinehuman TGF-1 ELISA; R&D Systems, Oxon, UK) with an assay range of 3.3–200 pg/mL [11].

(5) IGFBP-7 by the ELISA Kit (Ely, R&D Systems Inc., Abingdon, UK) with an assay range of 0.08–20 ng/mL [12].

### Statistical analysis

Data were analyzed using two-way analysis of variance followed by Dunnett’s test for post hoc multiple comparison. Results were expressed as mean, SDs, percentages, and correlation coefficient (*r*). For construction of interactive dot diagrams, RV and LV diastolic dysfunction were defined as a respective E/E′ratio >12 while RV and LV systolic dysfunction were defined as a respective GLS <18 according to guidelines, to assess the diagnostic accuracy of relevant biomarkers in detection of myocardial dysfunction [13,14]. The same cutoffs were used to compare the relevant biomarkers using area under the curve (AUC), net reclassification index (NRI), and integrated discrimination index (IDI) among diabetic patients.

## Results

This is a case-control study conducted on children with type 1 diabetes mellitus and other normal children according to Abu El Reesh Pediatric Hospital protocol.

Children were divided into two groups: (a) diabetic group (cases group) included 55 child with type 1 diabetes mellitus. They were 26 males (47.3%) and 29 females (52.7%). Their ages ranged between 6 and 12 years with a mean 9.6 ± 2.2. Non-diabetic group (control group) included 55 normal children. They were 28 males (50.9%) and 27 females (49.0%). Their ages ranged between 6 and 12 years with a mean 8.7 ± 1.7.

There is no statistical difference between the two groups regarding weight, height, BSA and BMI. HbA1c is statistically higher among diabetic group (Table 1).

**Table 1:** Demographic characteristics of cases of type 1 diabetes mellitus and controls

Variable	Type 1 diabetes mellitus (*n* = 55) Mean ± SD	Control (*n* = 55) Mean ± SD	*P* value^a^
Age (years)	9.6 ± 2.0	8.6 ± 1.7	0.07
Gender (F/M)	29/26	27/28	0.62
Weight (kg)	35.2 ± 9.4	37.7 ± 7.8	0.28
Height (cm)	133.0 ± 12.9	135.0 ± 10.0	0.298
BMI (kg/m^2^)	19.9 ± 2.3	19.0 ± 1.7	0.54
BSA (m^2^)	1.13 ± 0.27	1.12 ± 0.20	0.32
Diabetes mellitus duration (years)	4.5 ± 1.4	–	–
Glycated hemoglobin A1c (%)	9.6 ± 1.7	4.7 ± 0.5	<0.0001
LDL (mg/dL)	142 ± 4	138 ± 11	0.09
Triglycerides (mg/dL)	98 ± 5	77 ± 5	0.04
HDL (mg/dL)	68 ± 6	72 ± 4	0.77

Data are mean ± SD or ratio.

CI, confidence interval; HDL, high-density lipoprotein; LDL, low-density lipoprotein; –, non-applicable.

a Unpaired *t*-test unless otherwise specified.

Regarding echocardiographic measures of LV function: LV E′/A′ is significantly lower among cases compared to control group. LV E/E′ is significantly higher among cases compared to control group. LV Tei index is significantly higher among cases compared to control group. 3D LV GLS (%) is significantly lower among cases compared to control group. There is no statistically significant difference between the two groups regarding the 3D LV EF (Table 2).

**Table 2:** Echocardiographic measures of left ventricular function in cases of type 1 diabetes mellitus and controls

Variable	Type 1 diabetes mellitus (*n* = 55) Mean ± SD	Control (*n* = 55) Mean ± SD	*P* value^a^
LV E/E′	9.49 ± 2.78	6.87 ± 1.99	<0.0001
LV Tei index	0.524 ± 0.137	0.391 ± 0.088	<0.0001
3D LV GLS (%)	15.0 ± 4.3	20.9 ± 1.7	<0.0001
3D LV EDV (mL)	76.8 ± 18.6	72.5 ± 13.3	0.296
3D LV EF (%)	60.4 ± 4.3	58.5 ± 3.5	0.050
RV E/E′	7.90 ± 3.54	6.03 ± 3.12	0.024
RV Tei index	0.511 ± 0.173	0.418 ± 0.131	0.017
3D RV GLS (%)	14.8 ± 4.5	20.2 ± 3.0	<0.0001

Data are mean and SD.

3D, three dimensional; CI, confidence interval; LV EDV, left ventricular end diastolic volume; LV E/E′, ratio of early mitral inflow velocity to average early mitral annular and basal septal diastolic velocities; LV EF, left ventricular ejection fraction; LV GLS, left ventricular global longitudinal strain; RV E/E′, ratio of early tricuspid inflow velocity to early diastolic tricuspid annular velocity; RV GLS, right ventricular global longitudinal strain.

a Unpaired *t*-test.

On the other hand, echocardiographic measures of RV function: RV E′/A′ is significantly lower among cases compared to control group. RV E/E′ is significantly higher among cases compared to control group. RV Tei index is significantly higher among cases compared to control group. 3D RV GLS (%) is significantly lower among cases compared to control group (Table 2).

Comparing serum biomarkers between the two groups, BNP, CT-1, TGF-β, and IGFBP-7 are statistically higher among diabetic group. There is no statistically significant difference regarding ACV-A between the two groups (Table 3).

**Table 3:** Comparison of biomarkers in cases of type 1 diabetes mellitus and controls

Variable	Type 1 diabetes mellitus (*n* = 55) Mean ± SD	Control (*n* = 55) Mean ± SD	*P* value^a^
BNP (pg/mL)	169.9 ± 231.1	56.0 ± 19.5	0.015
ACV-A (pg/mL)	169.2 ± 213.3	96.2 ± 33.6	0.088
CT1 (pg/mL)	134.6 ± 68.6	55.1 ± 19.4	0.024
TGF-β (pg/mL)	209.1 ± 169.1	72.9 ± 72.4	0.0002
IGFBP-7 (ng/mL)	116.0 ± 80.9	34.7 ± 23.4	<0.0001

Data are mean and SD.

ACV-A, human activin A; BNP, brain natriuretic peptide; CI, confidence interval; CT-1, plasma cardiotrophin; IGFBP-7, human insulin-like growth factor binding protein-7; TGF-β1, human transforming growth factors β1.

a Unpaired *t*-test.

The relevant biomarkers which achieved statistically significant difference between cases and controls, namely BNP, CT-1, TGF- β, and IGFBP-7 were compared using NRI, IDI, and AUC (Table 4) for each of the LV systolic and diastolic function and similarly for the RV, respectively. BNP was the best biomarker for the diastolic function of the RV and LV while CT-1 was the best biomarker for LV systolic function and IGFBP-7 was the best predictor for RV systolic dysfunction.

**Fig. 1:**
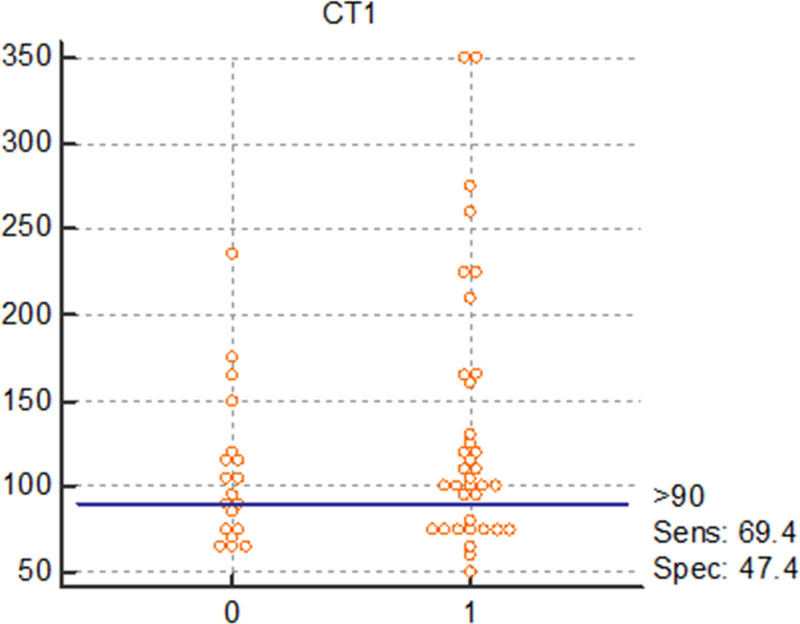
Interactive dot diagram for the diagnostic accuracy of CT-1 in diagnosis of LV systolic dysfunction as expressed by LV GLS. 0, Absence of systolic LV systolic dysfunction; 1, presence of LV systolic dysfunction according to definitions mentioned in statistical analysis section.

**Fig. 2:**
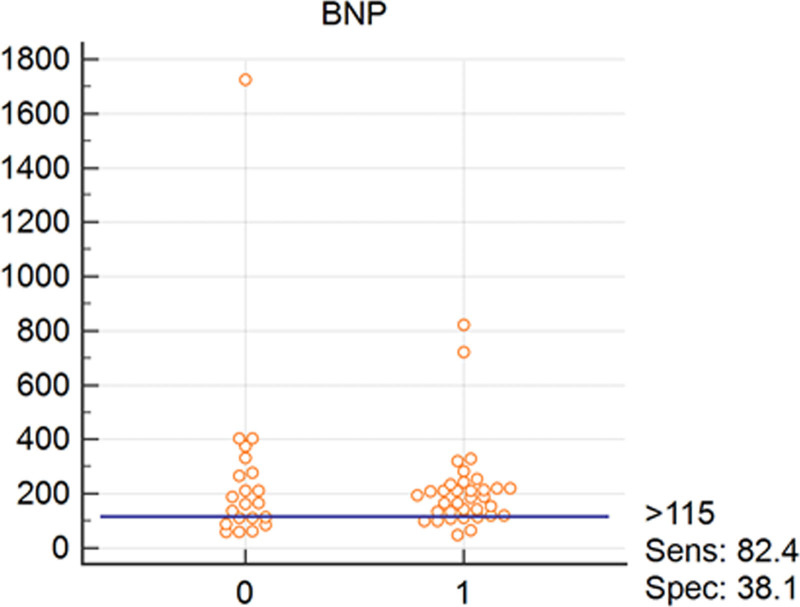
Interactive dot diagram for the diagnostic accuracy of BNP in diagnosis of LV diastolic dysfunction as expressed by LV E/E′. 0, Absence of diastolic LV systolic dysfunction; 1, presence of LV diastolic dysfunction according to definitions mentioned in statistical analysis section; BNP, brain natriuretic peptide.

**Fig. 3:**
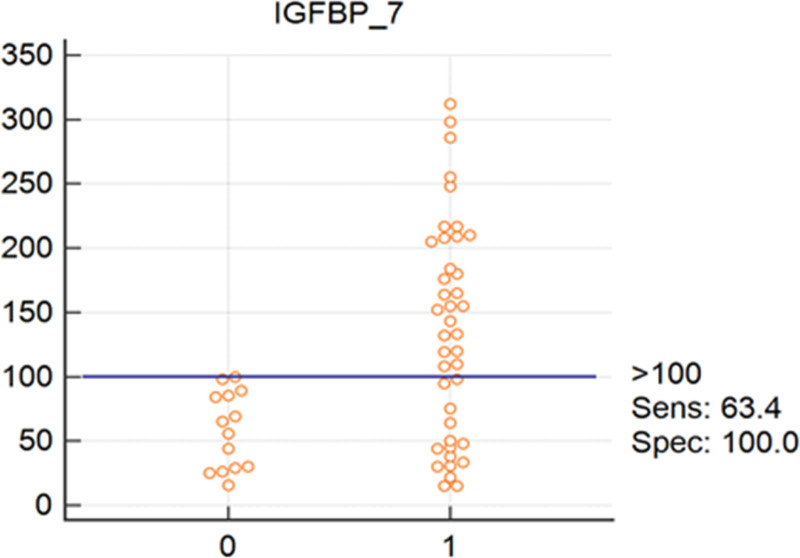
Interactive dot diagram for the diagnostic accuracy of IGFBP-7 in diagnosis of RV systolic dysfunction as expressed by RV GLS. 0, Absence of systolic RV systolic dysfunction; 1, presence of RV systolic dysfunction according to definitions mentioned in statistical analysis section; IGFBP, insulin-like growth factor binding protein-7.

**Fig. 4:**
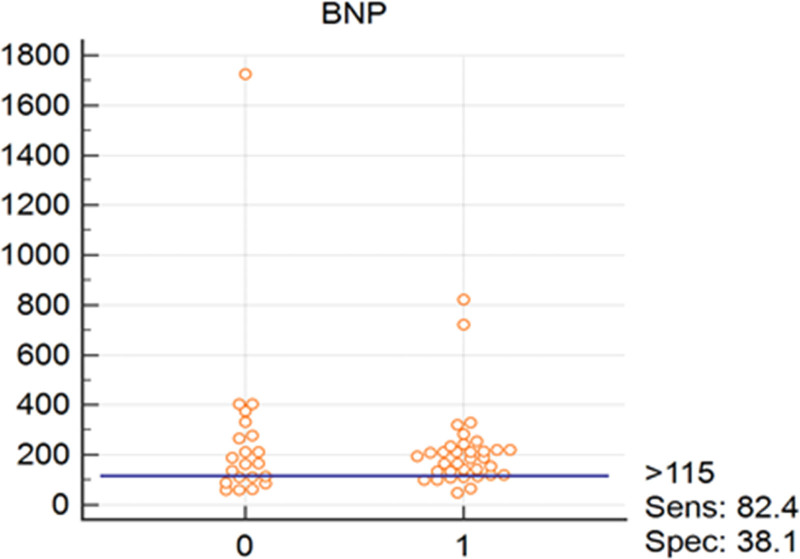
Interactive dot diagram for the diagnostic accuracy of BNP in diagnosis of LV diastolic dysfunction as expressed by RV E/E′. 0, Absence of diastolic RV systolic dysfunction; 1, presence of RV diastolic dysfunction according to definitions mentioned in statistical analysis section; BNP, brain natriuretic peptide.

Interactive dot diagrams have been designed to illustrate the diagnostic accuracy of each of the relevant biomarkers in diagnosis of RV and LV systolic and diastolic involvement. Figures 1 and 2 reflect the diagnostic accuracy of CT1 and BNP in prediction of systolic and diastolic LV involvement with sensitivity of 69 and 82%, respectively. While Figs. 3 and 4 represent the diagnostic accuracy of IGFBP 7 and BNP in predicting systolic and diastolic RV involvement with a sensitivity of 63 and 82 %, respectively.

## Discussion

New echocardiographic tools have offered important insights into the analysis of myocardial functions. They have the ability to detect subtle myocardial dysfunction before the development of overt symptoms and even before the appearance of gross changes in the conventionally used parameters such as M-Mode-derived EF and FS. This fact goes in agreement with results of our study which demonstrated an early affection of both systolic and diastolic functions in asymptomatic diabetic patients. Despite this confirmed accuracy, the performance of tedious and refined echocardiographic techniques such as speckle-tracking echocardiography (STE) and TDI requires experienced operators in image acquisition and subsequent analysis. Also, the scanning and interpretation times are long due to the complexity of the data offered by analyzing software [15].

The aforementioned facts raise the need for readily available and reliable serum markers that can be used as a diagnostic modality till results of echocardiography are available and also for regular follow up of the patient even in the absence of echocardiographic examination.

Diabetes mellitus offers a good biologic model for testing the accuracy of several old and new biomarkers against benchmark parameters of advanced TDI and STE. Diabetes mellitus is known to impact the heart due to reliance of the heart on fructose metabolism and subsequent progressive cardiomyocyte degeneration that is independent of glycemic control. This process of progressive myocardial involvement offers a good background to test the cardiac biomarkers not only to test their accuracy but also to understand the possible pathogenesis and cellular mechanisms contributing to the process of myocardial degeneration in this systemic disease [2].

In our study, CT-1 proven to be the best predictor of LV systolic dysfunction. CT-1 is secreted in cardiac fibroblasts; in acute stress, CT-1 promotes cell survival. However, if stressful signals persist, chronic upregulation of CT-1 leads to cardiomyocyte hypertrophy and, finally, LV dysfunction. The specificity of CT-1 in systolic heart failure has been proven by Freed *et al*. [16] in post-MI sequelae. Its elevation has been linked to the persistent systolic dysfunction after the development of myocardial scar [17].

Only one study showed the correlation between CT-1 and newly diagnosed diabetes mellitus in adults from the Chinese population [18]. Our study is a novel one studying CT-1 in children with type 1 diabetes mellitus and its correlation with diabetic cardiomyopathy.

There is scarcity of data regarding RV dysfunction in diabetics. Most of the studies are focused on LV deformation. In our study, patients displayed evidence of RV diastolic and systolic dysfunction in the form of prolonged Tei index and decreased GLS, respectively. The RV is usually jeopardized by volume load. It is increasingly recognized that even non-complicated diabetes is accompanied with a state of increased preload. This is due to overall increase of exchangeable sodium content with subsequent increase in fluid retention in the extracellular fluid [19].

As per markers of RV systolic dysfunction, statistical multiple regression analysis test for IGFBP-7 was found to have the highest statistically significant predictive factor (*P* < 0.0001) with a cutoff predictive value of ≥100 ng/mL (sensitivity 64% and specificity 100%).

The diagnostic accuracy of IGFBP-7 has been mainly investigated in the context of the LV. Motiwala *et al*. [20] assessed in his series the LV remodeling and cardiac events in patients with heart failure among several outpatients’ office visits in 142 patients using five different biomarkers with biologic links to cardiac remodeling. Their results showed that increased levels of IGFBP-7 were mostly associated with cardiac events while lowest levels predicted fewer events (*P* = 0.01). Moreover, subjects with higher IGFBP-7 had decreased diastolic function LV E/E′ (*P* = 0.07) and they suggested that serial measurements of IGFBP-7 provide a prognostic information regarding myocardial remodeling. There is scarcity of data about the usefulness of IGFBP-7 in assessing RV functions. Gandhi *et al*. [21] showed a correlation between IGFBP-7 and RV systolic pressure.

In contrast, BNP was found to be a good marker of diastolic dysfunction whether in RV or LV. This goes in agreement with several reports denoting that the release of BNP is linked to LV stretch and raised diastolic pressures of the ventricles. This makes it a better marker for diastolic dysfunction and HFpEF than any of the other tested markers. Its increase in use over the last years has made its availability in the emergency and outpatient setting possible [3].

Many studies were done on BNP and cardiac function in patients with heart failure and renal failure on hemodialysis or patients being prepared for heart transplant. They all found that BNP correlated with LV ejection fraction and LV E/E and it is a good indicator of diastolic dysfunction in asymptomatic patients [22–24].

Similarly, Salem *et al*. [25] found pro-BNP significantly elevated among children and adolescence with type 1 diabetes mellitus (*P* < 0.01) and the impaired diastolic function was related to the control of diabetes. They concluded that asymptomatic diabetics had evidence of subtle RV and LV dysfunction with delayed myocardial relaxation which was related to metabolic control.

### Conclusion

We are living in the era of cardiac biomarkers, and in this evolving era, it is not only important to use them but to understand which of them can point towards a specific pattern or specific pathogenesis. CT-1 has proven to be a diagnostic superiority in LV systolic dysfunction whilst BNP continue to prove everyday through our study and through many other studies that it is the chief marker of diastolic dysfunction and HFpEF. This potential accuracy and the increasing availability of BNP in the outpatient setting makes it clear that it should be used as a screening test not only in diabetic patients and as part of their regular follow-up but also in every systemic disease predisposing to myocardial involvement.

**Table 4:** Biomarkers of left ventricular systolic function

	NRI		IDI		AUC
	Event	Non-event	Event	Non-event	
CT-1	−0.182 (−0.396, 0.321)	0.455 (0.128, 0.688)	0.057 (−0.004, 0.182)	0.037 (−0.003, 0.112)	0.7632 (0.0042)
BNP	0.182 (−0.380, 0.619)	0.636 (0.122, 0.836)	0.075 (−0.005, 0.253)	0.048 (−0.010, 0.176)	0.7062 (0.0004)
IGFBP-7	0.182 (−0.372, 0.522)	0.212 (−0.241, 0.556)	0.025 (−0.016, 0.146)	0.016 (-0.010, 0.094)	0.5937 (0.0157)
TGF-β1	0.262 (−0.142, 0.378)	0.599 (−0.051, 0.634)	0.117 (−0.07, 0.231)	0.212 (−0.02, 0.321)	0.459 (0.08)

AUC, area under the curve; BNP, brain natriuretic peptide; CT-1, plasma cardiotrophin; IDI, integrated discrimination index; IGFBP-7, human insulin-like growth factor binding protein-7; NRI, net reclassification index; TGF-β1, human transforming growth factors β1.

**Table 5:** Biomarkers of left ventricular diastolic function

	NRI		IDI		AUC
	Event	Non-event	Event	Non-event	
CT-1	−0.353 (−0.500, 0.533)	0.333 (−0.647, 0.745)	0.005 (−0.016, 0.044)	−0.002 (−0.026, 0.082)	0.5231 (0.6031)
BNP	−0.294 (−0.383, 0.389)	−0.429 (−0.631, 0.625)	−0.014 (−0.015, 0.053)	−0.033 (−0.040, 0.090)	0.7777 (0.1603)
IGFBP-7	0.000 (−0.351, 0.375)	0.333 (−0.600, 0.571)	−0.002 (−0.014, 0.047)	−0.004 (−0.026, 0.080)	0.5602 (0.3632)
TGF-β1	0.163 (0.013, 0.321)	0.231 (0.003, 0.343)	0.278 (0.013, 0.311)	0.211 (0.07, 0.381)	0.381 (0.231)

AUC, area under the curve; BNP, brain natriuretic peptide; CT-1, plasma cardiotrophin; IDI, integrated discrimination index; IGFBP-7, human insulin-like growth factor binding protein-7; NRI; net reclassification index; TGF-β1, human transforming growth factors β1.

**Table 6:** Biomarkers of right ventricular systolic function

	NRI		IDI		AUC
	Event	Non-event	Event	Non-event	
CT-1	−0.294 (−0.464, 0.485)	0.143 (−0.700, 0.800)	−0.007 (−0.016, 0.043)	−0.009 (−0.024, 0.076)	0.5938 (0.4091)
BNP	0.294 (−0.398, 0.500)	−0.143 (−0.474, 0.444)	−0.001 (−0.020, 0.052)	−0.020 (−0.029, 0.088)	0.5175 (0.9604)
IGFBP-7	−0.235 (−0.377, 0.429)	0.143 (−0.636, 0.631)	−0.011 (−0.013, 0.053)	−0.012 (−0.028, 0.092)	0.7875 (0.0144)
TGF-β1	0.289 (−0.317, 0.341)	0.344 (−0.218, 0.422)	0.044 (−0.021, 0.235)	0.214 (0.114, 0.321)	0.419 (0.331)

AUC, area under the curve; BNP, brain natriuretic peptide; CT-1, plasma cardiotrophin; IDI, integrated discrimination index; IGFBP-7, human insulin-like growth factor binding protein-7; NRI, net reclassification index; TGF-β1, human transforming growth factors β1.

**Table 7:** Biomarkers of right ventricular diastolic function

	NRI		IDI		AUC
	Event	Non-event	Event	Non-event	
CT-1	−0.217 (−0.538, 0.362)	0.500 (−0.389, 0.754)	0.027 (-0.013, 0.158)	0.023 (−0.018, 0.115)	0.6664 (0.0159)
BNP	−0.043 (−0.470, 0.619)	0.625 (0.233, 0.809)	0.042 (−0.008, 0.207)	0.037 (−0.013, 0.160)	0.7473 (0.002)
IGFBP-7	−0.217 (−0.545, 0.500)	−0.375 (−0.419, 0.352)	−0.002 (−0.014, 0.057)	−0.014 (−0.020, 0.045)	0.5224 (0.4676)
TGF-β1	0.032 (−0.343, 0.522)	0.255 (−0.213, 0.311)	0.122 (−0.033, 0.154)	0.237 (−0.052, 0.281)	0.422 (0.422)

AUC, area under the curve; BNP, brain natriuretic peptide; CT-1, plasma cardiotrophin; IDI, integrated discrimination index; IGFBP-7, human insulin-like growth factor binding protein-7; NRI, net reclassification index; TGF-β1, human transforming growth factors β1.

## Acknowledgements

**Conflicts of interest**

There are no conflicts of interest.
